# Differential transcriptomics in sarcoidosis lung and lymph node granulomas with comparisons to pathogen-specific granulomas

**DOI:** 10.1186/s12931-020-01537-3

**Published:** 2020-12-04

**Authors:** Nancy G. Casanova, Manuel L. Gonzalez-Garay, Belinda Sun, Christian Bime, Xiaoguang Sun, Kenneth S. Knox, Elliott D. Crouser, Nora Sammani, Taylor Gonzales, Bhupinder Natt, Sachin Chaudhary, Yves Lussier, Joe G. N. Garcia

**Affiliations:** 1 Department of Medicine, College of Medicine, University of Arizona Health Sciences, Tucson, AZ USA; 2 Department of Medicine, College of Medicine, University of Arizona, Phoenix, AZ USA; 3 Division of Pulmonary and Critical Care Medicine, The Ohio State University, Columbus, OH USA

**Keywords:** Sarcoidosis, Valley fever, Tuberculosis, Gene expression, Granulomatous, Biomarker

## Abstract

**Rationale:**

Despite the availability of multi-“omics” strategies, insights into the etiology and pathogenesis of sarcoidosis have been elusive. This is partly due to the lack of reliable preclinical models and a paucity of validated biomarkers. As granulomas are a key feature of sarcoidosis, we speculate that direct genomic interrogation of sarcoid tissues, may lead to identification of dysregulated gene pathways or biomarker signatures.

**Objective:**

To facilitate the development sarcoidosis genomic biomarkers by gene expression profiling of sarcoidosis granulomas in lung and lymph node tissues (most commonly affected organs) and comparison to infectious granulomas (coccidiodomycosis and tuberculosis).

**Methods:**

Transcriptomic profiles of immune-related gene from micro-dissected sarcoidosis granulomas within lung and mediastinal lymph node tissues and compared to infectious granulomas from paraffin-embedded blocks. Differentially-expressed genes (DEGs) were profiled, compared among the three granulomatous diseases and analyzed for functional enrichment pathways.

**Results:**

Despite histologic similarities, DEGs and pathway enrichment markedly differed in sarcoidosis granulomas from lymph nodes and lung. Lymph nodes showed a clear immunological response, whereas a structural regenerative response was observed in lung. Sarcoidosis granuloma gene expression data corroborated previously reported genomic biomarkers (*STAB1, HBEGF,* and *NOTCH4*), excluded others and identified new genomic markers present in lung and lymph nodes, *ADAMTS1, NPR1 and CXCL2*. Comparisons between sarcoidosis and pathogen granulomas identified pathway divergences and commonalities at gene expression level.

**Conclusion:**

These findings suggest the importance of tissue and disease-specificity evaluation when exploring sarcoidosis genomic markers. This relevant translational information in sarcoidosis and other two histopathological similar infections provides meaningful specific genomic-derived biomarkers for sarcoidosis diagnosis and prognosis.

## Introduction

Sarcoidosis is a multisystemic disease of unknown etiology characterized by the formation of granulomatous lesions, especially in lung tissues and thoracic lymph nodes [1, 2]. The clinical heterogeneity and unpredictable disease course in sarcoidosis represents a challenge in early diagnosis and prediction of progression. Despite significant progress in the understanding of genetic predisposition and role of immunity in sarcoidosis pathogenesis, predicting the clinical course of sarcoidosis in a given patient remains challenging. The majority of sarcoidosis patients experience spontaneous recovery, however, fully a third of subjects develop complicated sarcoidosis defined as multiorgan involvement, progressive fibrotic lung involvement and pulmonary function deterioration [3, 4]. Validated sarcoidosis biomarkers are desperately needed to distinguish patients who will spontaneously recover from patients who will worsen and develop severe manifestations of the disease. Thus, personalized medicine approaches are tasked with the capacity to predict the patient phenotype likely to develop progressive disease or that may eventually require lung transplantation. While there has been progress in identifying genetic variants that contribute to complicated sarcoidosis [5, 6], and we have previously utilized genomic expression profiling of peripheral mononuclear cells (PBMCs) to subphenotype patients with a variety of inflammatory disorders [7–9] including sarcoidosis [10], there remains an important and unmet need for molecular and specific genome-based biomarkers for diagnosis and prediction of disease severity in sarcoidosis.

The etiology of sarcoidosis is unknown, and the diagnosis remains one of exclusion [4]. Specific infections, including a variety of Mycobacterium species and Propionibacterium acnes [11–20], and environmental exposures [21] produce granulomatous inflammatory lesions that mimic sarcoidosis and have been posited as potential etiologic agents. Exposure to mycobacterium tuberculosis (TB) and endemic fungi such coccidioidomycosis (CM), also known as Valley Fever, are major causes of granulomatous lung disease [15] and must be clinically excluded when sarcoidosis is suspected. Coccidioidomycosis caused by *Coccidioides immitis* and *C. posadasii*, a soil-dwelling fungi disease, are endemic in the arid southwestern USA and northern Mexico.

Granulomas are conglomerates of epithelioid and multinucleated giant cells surrounded by CD4+ and CD8+ T lymphocytes that result from the complex immunopathogenesis between host genetic factors, environmental or infectious triggers [11]. Sarcoidosis array-based transcriptomic studies in blood, lung and lymph node, lacrimal gland, orbital tissue and skin identified tissue-specific differential gene expression [22–25], highlighting the importance of assessing different compartments in a multisystemic disease such as sarcoidosis. The majority of these transcriptome studies were focused on identifying gene signatures that differentiate controls from progressive forms of sarcoidosis. Additional studies in peripheral blood compared the gene expression of sarcoidosis and TB, identifying a significant overlap in gene expression associated with Type I and II IFN pathways [24]. Approaches to quantify the expression of sarcoidosis granuloma-related genes using RT-PCR focused solely on cytokine-related genes associated with NFKB and STAT transcription factors [26].

As expression profiling of sarcoidosis granuloma using next generation sequencing expression has not been reported, one goal of the present study was to compare gene expression profiles in two sarcoidosis granulomatous tissues: lung and lymph node. The second goal of this study was to assess the ability of granuloma gene expression profiles to discriminate sarcoidosis from TB and CM. Our bioinformatic analyses included determination of unique pathways associated with sarcoidosis and overlapping pathways with CM and TB. Our results indicate that despite histologic similarities, sarcoidosis lung and lymph node granulomas exhibit distinct expression profiles. In addition, sarcoidosis pathway expression was significantly divergent compared to TB and CM. These findings suggest the importance of tissue-specific pathobiology considerations to be employed when exploring potential sarcoidosis genomic markers for potential diagnostic use and discriminate from other granulomatous diseases.

## Materials and methods

### Sample selection and design

We used microdissected granulomatous tissue from lung [6] and mediastinal lymph node [12] from 18 subjects with sarcoidosis, three with CM, four with TB and control tissue from lungs [3] and lymph nodes [3] from 6 healthy individuals. Patient and sample characteristics are described in Table 1. Tissue specimens were collected from clinically-indicated biopsies for diagnostic purposes. Archived, de-identified specimens were acquired from the Tissue Acquisition Shared Resource at the University of Arizona. The study was approved by the human subjects protection program IRB # 1509097312A001.

**Table 1:** Patient and sample characteristics

Variable	Sarcoidosis	CM	TB
Number of cases	18	3	4
Granuloma origin			
Lymph node (mediastinal)	12	0	2
Lung	6	3	2
Sex, female, N (%)	66	33	50
Age, (mean, Sd)	52.8 ± 11.4	38.3 ± 16.5	58.3 ± 9.3
Race/ethnicity, N (%)			
Black or African American	11	0	0
White	62	100	100
Hispanic*	17	66	75
Other**	11	na	na

Granuloma specimens from 18 sarcoidosis, 3 Coccidiodomycosis (CM) and 4 tuberculosis (TB) patients were included in this study. There was no significant difference in age (p = 0.1). The predominant race was White in the three granulomatous diseases, 11% of the sarcoidosis were self-identified as Black or African Americans, while *half of Hispanics were self-identified as white as well. **Other include Native American and Multiracial

### Gene expression profiling of selected granulomas

Formalin-fixed, paraffin-embedded (FFPE) microdissections from lung and lymph node granulomatous tissue and healthy tissue (controls) were assayed using Next Generation Sequencing-based gene expression by HTG EdgeSeq Oncology-biomarker (HTG Molecular Diagnostics, Inc.). This system uses targeted capture sequencing to quantitate RNA expression levels of gene targets in FFPE tissues. The panel included 2535 probes and 15 housekeeper genes for quantitative analysis of targeted mRNAs. A list of the complete biomarker panel genes and the biological pathways assayed are in Additional file 1: Table S1. This panel covers multiple immune-oncologic-related pathways previously published and identified as relevant in sarcoidosis including such as interferon, MAP kinase, NFКB, and JAK/STAT pathways [18–20]. Representative tissue sections from granulomatous tissue were stained with hematoxylin- and eosin (H&E). Sites for granuloma microdissection were selected by an expert pathologist (Additional file 2). Sections of ~ 1.5 mm^2^ thickness of a 5 μm FFPE tissue were obtained, 5–10 sections per subject were lysed followed by RNA extraction-free chemistry method using the HTG^®^’s assay kit. Library preparation for the HTG Edge processor for nuclease protection steps was used, followed by PCR tagging, library amplification, quantitation and normalization. Library was then sequenced on an Illumina MiSeq using 150-cycle V3 kits [27].

### Analysis of biomarker transcriptomes and canonical pathways

We generated the transcriptome index using the salmon package [28] and a fasta file containing the sequence of the HTG EdgeSeq probes. Quantification was performed for each sample using the salmon quant command [28], the index file and the corresponding fastq files. The raw counts were loaded into R using the tximport package [29]. The Differential Expression (DE) was calculated using Limma [30] and EdgeR [31] packages from the R v3.5.3 and Bioconductor v3.8 packages [32]. A list with statistically-significant genes for each comparison was submitted for pathway enrichment unbiased comparison of the DE gene sets to the human ConsensusPathDB [33] website using the over-representation gene set analysis against the pathway databases and the gene ontology categories using a minimum overlap with input list of 2 and a p-value cutoff of 0.01.

### Transcriptome dataset validation

We validated our transcriptome results in three independent cohorts of microarray datasets publicly available in the Gene Expression Omnibus (GEO), GSE16538 and GSE63548 [34] and an unpublished microarray dataset, details in Additional file 3: Table S3. At the time this manuscript was written, no GEO data sets were available for humans infected with CM. The microarray datasets for TB and sarcoidosis were analyzed using R (v3.5.3) and Bioconductor (v3.8) [32]. Oligo package (v1.46.0) was used for the processing of the data in combination with specific platform design library and the corresponding annotation library. For the Affymetrix array analyses, Oligo’s implementation of the Robust Multichip Average algorithm (RMA) for background correction, quantile normalization and summarization was used. For the Illumina arrays, the Illuminaio package [35] was used in combination with the corresponding bead Chip. The differential expression analysis was performed using the Limma package [36]. After normalization gene expression was averaged and each tissue was compared against heathy tissue from the same origin. The inverse log2 (FC) generated by Limma was used to determine differences between comparisons, with the direction of change indicated by the sign. Transcripts with a fold change (FC) > 2 and a p value < 0.01 were deemed differentially expressed.

## Results

### Patient characteristics

Microdissected lung and lymph node granulomas from 18 sarcoidosis subjects, four TB subjects, three CM subjects and six healthy controls were assessed. There were no significant differences in age (p < 0.1) in the three granulomatous disease groups (Table 1). The majority of sarcoidosis subjects were female and non-Hispanic whites while the demographic most frequent reported in the CM and TB groups was Hispanic. Clinical data related to organ involvement and radiographic Scadding stage was available for 70% of sarcoidosis subjects as detailed in Additional file 1: Table S1.

**Fig. 1:**
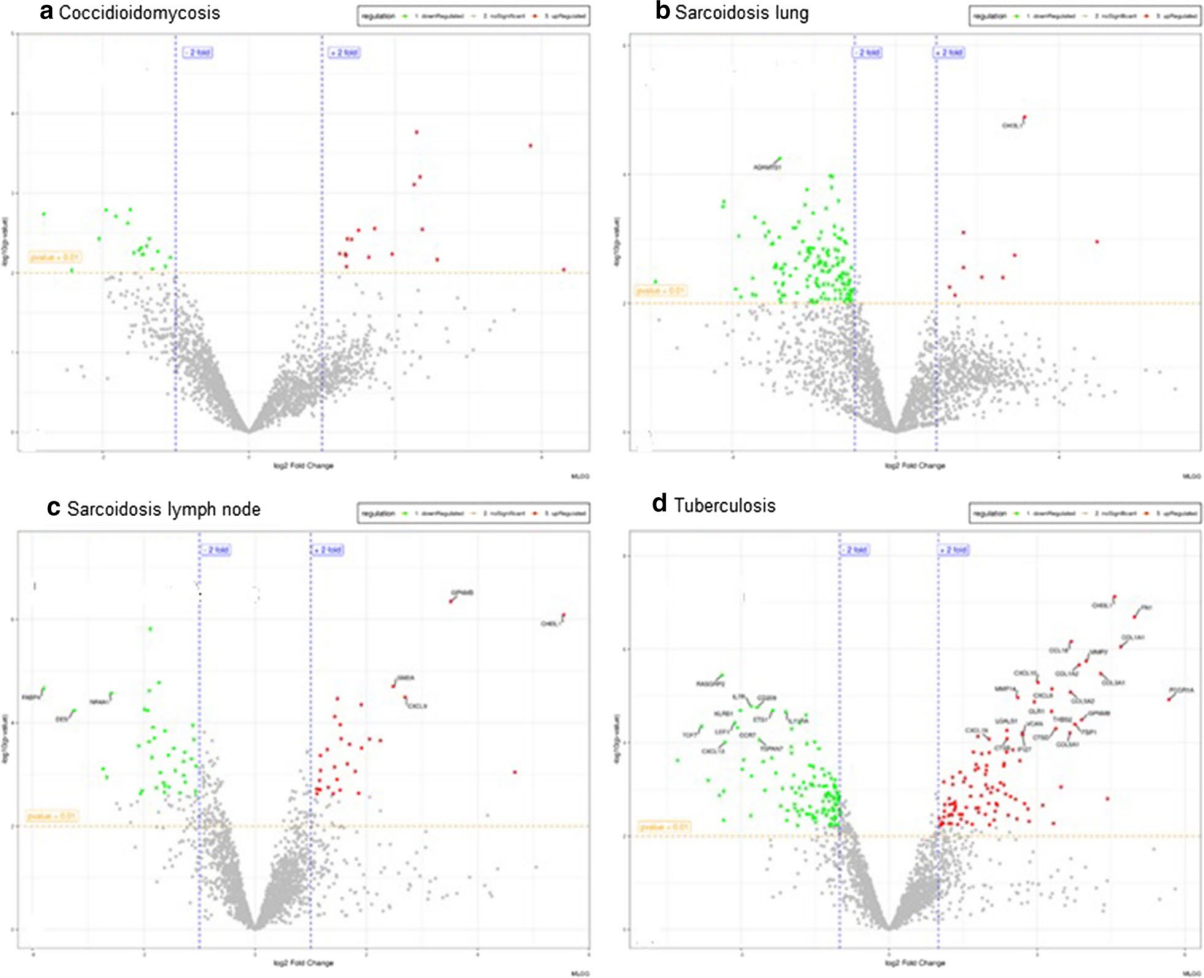
Results of the differential expression analysis. Volcano plot of overall gene-based differential expression. Comparisons were disease and tissue specific vs healthy controls. The x-axis corresponds to the log(base2) of the fold change difference and the y-axis corresponds to the negative log(base10) of the p-values. Downregulated transcripts are indicated in green and upregulated transcripts in red. **a** Lung cocci, **b** Lung Sarcoidosis, **c** Lymph Sarcoidosis, **d** Lymph TB

**Fig. 2:**
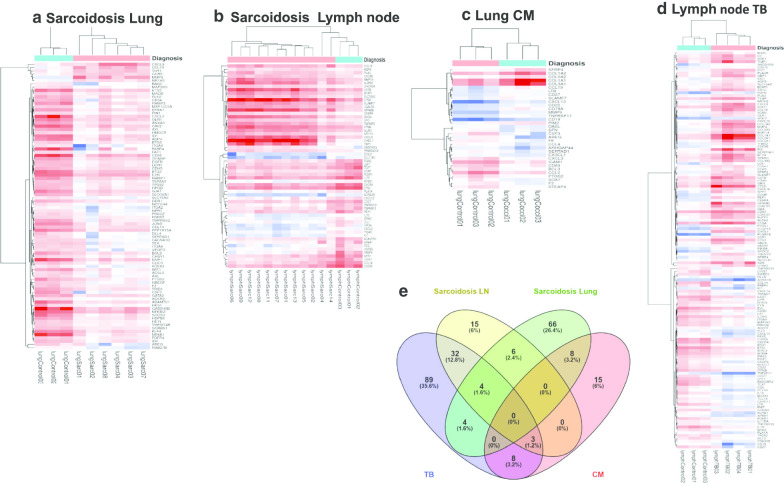
Heatmaps representing the expression profiles of the DEG from granulomatous tissue against healthy controls. **a** Sarcoidosis lung granulomas, **b** Sarcoidosis lymph node granulomas, **c** Coccidioidomycosis lung granulomas, **d** Tuberculosis lymph node granulomas. Red indicates decreased expression; blue indicates increased expression. **e** Venn Diagram analysis representing the 250 total DEGs with the overlapping and unique differentially expressed transcripts between granulomas from sarcoidosis (lymph node and lung), TB (lymph node) and CM (lung)

After excluding probes with multiple hits or partial hits, our gene expression data in granulomatous tissues included 2430 probes that uniquely mapped to single genes. We averaged gene expression levels after normalization and compared diseased groups versus healthy tissue controls from the same tissue origin, the fold change (FC) was obtained as the ratio between the averages. We identified 250 significantly dysregulated genes between all granulomatous-diseased tissues and healthy control tissues. Figure 1 represents the differentially expressed genes (DEGs) volcano plots, in each category; granulomas from CM exhibited the fewest number of differentiated transcripts, 34 in total (Fig. 1a), sarcoidosis granulomas from the lung (Fig. 1b) had more DEGs than those granulomas from lymph nodes (Fig. 1c), 88 vs 60 dysregulated transcripts respectively. Notably, in both sarcoidosis-affected tissues, the number of downregulated genes (FC < − 2) outpaced the number of upregulated genes (FC > 2), with downregulation more marked in lung granulomas (93% of the transcripts) than in lymph node granulomas (56% of the transcripts). Tuberculosis granulomas (Fig. 1d), showed the highest number of the differentiated transcripts, 140 in total. The heat maps comparing the gene expression in each group against healthy tissues are presented in Fig. 2. In addition, we screened the total of 250 DEGs among those four sets performing a Venn diagram analysis [37]. Figure 2e shows the overlapping and unique transcripts in the different granulomas. Sarcoidosis granulomas exhibited 138 dysregulated transcripts in both lung (Fig. 2a) and lymph node (Fig. 2b), 87 transcripts were exclusive present in sarcoidosis, notoriously, 30% of the DEGs in lung granulomas were unique to sarcoidosis, and the overlapping transcripts dysregulated in both sarcoidosis tissues was only 4%. TB had 89 DEG that were exclusively dysregulated in TB granulomas. When compared TB to sarcoidosis 17% of the transcripts were dysregulated in both diseases, while only 4% of the transcripts were common between CM and lung sarcoidosis.

### Comparisons of expression profiles in sarcoidosis granulomas

**Fig. 3:**
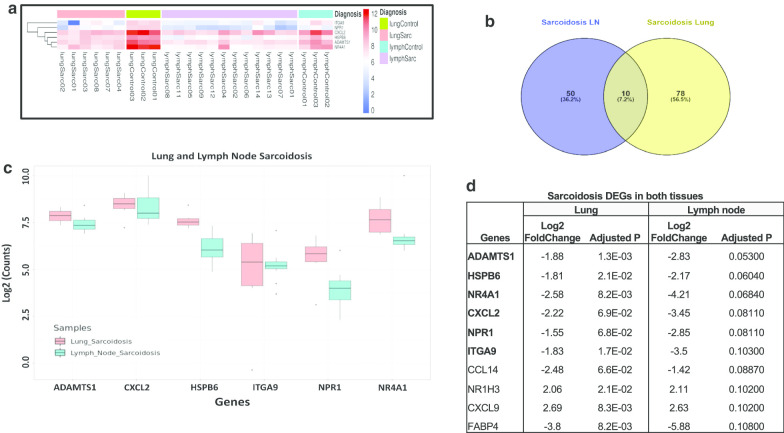
Sarcoidosis dysregulated genes present in lung and lymph node. **a** Heatmap representing the expression of the six DEGs only present in granulomas from sarcoidosis lung and lymph node. **b** Vendiagram showing the dysregulated transcripts in both granulomatous sarcoidosis tissues (lungs and lymph nodes). **c** Box plot of the six transcripts dysregulated in lung and lymph nodes from sarcoidosis. Y axis represents the Log 2 counts for each transcript. **d** Transcripts dysregulated in lung and lymph node. Six genes in bolded where present exclusively in sarcoidosis the last four were also significantly dysregulated in TB

We identified that sarcoidosis granulomas from lung and lymph nodes share only ten DEGs. *NR1H3* and *CXCL9* were upregulated whereas the remainder (*ADAMTS1, CXCL2, HSPB6, ITGA9, NPR1, NR4A1, CCL14, FABP4*) showed downregulation in both lung and lymph nodes. Interrogation of the expression levels of these 10 sarcoidosis genes to TB and CM DEGs revealed *CCL14, CXCL9, FABP4, NR1H3* were also significantly dysregulated in TB. Only six genes (*ADAMTS1, HSPB6, NR4A1, CXCL2, NPR1, ITGA9*) were exclusively dysregulated in both lung and lymph node in sarcoidosis granulomas but not in CM or TB (Fig. 3). These results are very consistent with the notion that granuloma gene expression is highly tissue- and disease-specific.

### Comparison of DEGs in sarcoidosis vs CM and TB granulomas

Comparison of TB lymph node granulomas expression profiles to lymph node healthy controls identified 140 DEGs with a surprising number of DEGs common to sarcoidosis, 43 transcripts, 39 present in lymph granulomas. Unlike sarcoidosis where DEGs were predominantly downregulated, TB DEGs showed a balanced proportion of expression, 64 down-regulated and 76 upregulated. Notably, we observed the same direction in the transcript regulation, in all the 43-shared transcripts, except for one, *OLR1*, a low density lipoprotein receptor involved in Fas-induced apoptosis, this gene was upregulated in TB and down regulated in sarcoidosis, over-expression of *OLR1* result in upregulation of NF-κB and its target pro-oncogenes [38].

Gene expression profiles from CM lung granulomas exhibited the fewest number of dysregulated genes, 34 DEGs with a balanced pattern of dysregulation, 18 DEGs downregulated, 16 DEGs upregulated. A total of 15 DEGs were exclusively dysregulated in CM granulomas. Eight upregulated transcripts were common to sarcoidosis lung granulomas (*CSF3, SERTAD1, MMP9, CCL19, BCL3, AREG, PTGS, F3*). Only three DEGs, *SLAMF7, DC27,* and *CXCL13*, were dysregulated in all three diseases (sarcoidosis, TB, CM). Expression of *CXCL13*, a chemokine and B-lymphocyte chemoattractant associated to calcium influx was downregulated in both sarcoidosis (lymph node) and TB granulomas but upregulated CM granulomas. A list of the top dysregulated genes by granuloma tissue origin is available in Additional file 5: Table S4.

### Functional biologic pathway enrichment analysis

**Fig. 4:**
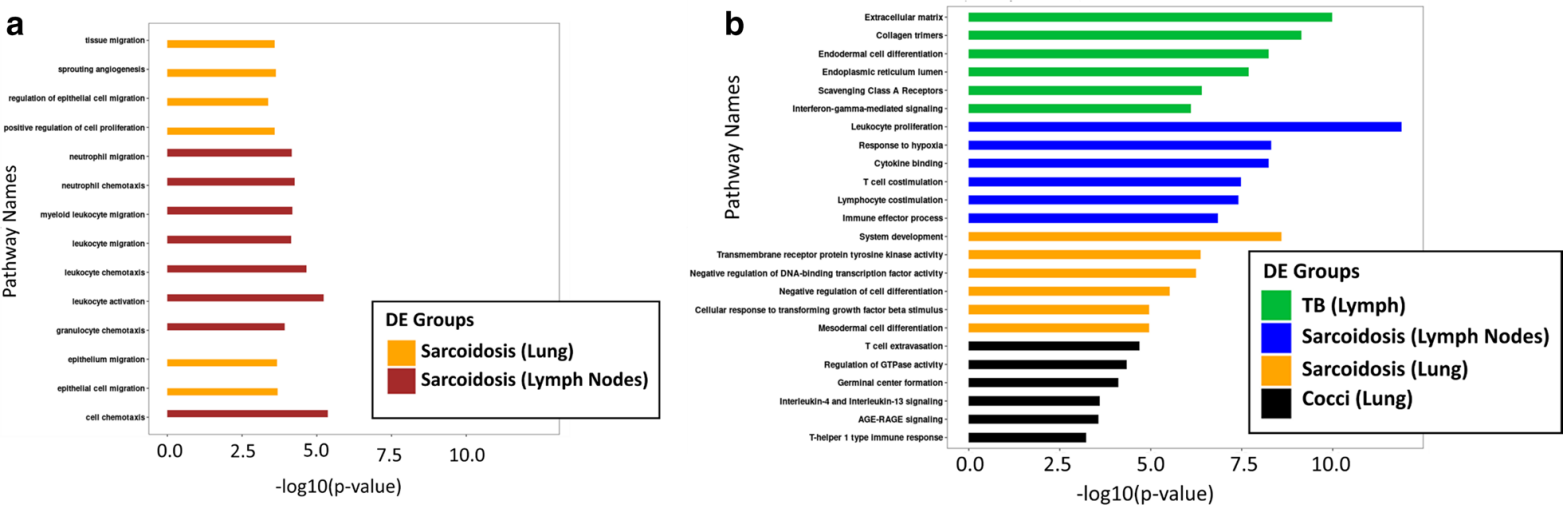
Functional Enrichment Analysis. Top over representations of DEGs against the pathway databases and the gene ontology. The x- axis corresponds to the negative log (base 10) of the p-values generated by ConsensusPathDB. **a** Sarcoidosis different pathways contrasting the gene expression on granulomas from lung (yellow) and Lymph nodes (red). **b** Differences among the top pathways enriched in each granulomatous tissue by disease category. TB (green), Sarcoidosis lymph node (blue), Sarcoidosis lung (yellow) CM (black). Upregulated genes in red and downregulated genes in blue

We first identified the pathways that were common to all three granulomatous diseases using the gene list with all the DEG (250), cytokine-cytokine receptor interaction, chemokine signaling, VEGFA-VEGFR2 and focal adhesion accounted for the most significant pathways with the most number of proportionally dysregulated genes involved, we then mapped the pathways associated to the genes that were exclusively present in sarcoidosis. Sarcoidosis pathways showed similar altered pathways to the rest of the granulomatous diseases with the exception of nuclear receptors meta-pathway (Table 2). Then we compared the differences in pathways between lung and lymph nodes in sarcoidosis, using the gene set of DEG for each tissue, differences were delineated by a clear immunological response, involving leucocyte migration and neutrophil chemotaxis in the lymph nodes while a structural regenerative response, characterized by cell migration and angiogenesis, was observed at the lung level (Fig. 4a). At the disease level, we identified pathways that were disease specific for each gene set as seen in Fig. 4b.

**Table 2:** Pathway analysis

Pathway name	Gene set size	Genes contained	p-value	q-value	Source
Granulomatous tissue					
Cytokine-cytokine receptor interaction	294	37 (12.6%)	1.5E−20	4.2E−19	KEGG
Chemokine signalling pathway	189	24 (12.7%)	9.8E−14	1.4E−12	KEGG
VEGFA-VEGFR2 Signaling Pathway	236	26 (11.0%)	2.6E−13	2.4E−12	Wikipathways
PI3K-Akt signalling pathway	354	31 (8.8%)	5.6E−13	3.9 E−12	KEGG
Focal Adhesion-PI3K-Akt-mTOR-signaling	302	28 (9.3%)	2.2E−12	1.2E−11	Wikipathways
PI3K-Akt Signaling Pathway	340	29 (8.6%)	6.4E−12	3E−11	Wikipathways
Pathways in cancer	526	36 (6.8%)	1.1E−11	4.4E−11	KEGG
Focal Adhesion	198	22 (11.1%)	1.7E−11	5.8E−11	Wikipathways
Extracellular matrix organization	294	26 (8.8%)	4.1E−11	1.3E−10	Reactome
Focal adhesion	199	21 (10.6%)	1.3E−10	3.6E−10	KEGG
Human papillomavirus infection	339	23 (6.8%)	8.9E−08	2.3E−07	KEGG
Signal Transduction	2647	82 (3.1%)	4.1E−07	9.1E−07	Reactome
Cytokine Signaling in Immune system	458	26 (5.7%)	4.2E−07	9.1E−07	Reactome
Immune System	1840	63 (3.4%)	5.2E−07	1E−06	Reactome
MAPK signalling pathway	295	20 (6.8%)	6.5E−07	1.2E−06	KEGG
JAK STAT pathway and regulation	310	20 (6.5%)	1.3E−06	2.3E−06	INOH
Signaling by Receptor Tyrosine Kinases	423	21 (5.0%)	4.3E−05	7.1E−05	Reactome
GPCR ligand binding	466	22 (4.7%)	5.8E−05	9.1E−05	Reactome
Hemostasis	668	25 (3.7%)	0.00069	0.00101	Reactome
Adaptive Immune System	732	25 (3.4%)	0.00232	0.00324	Reactome
Innate Immune System	1077	31 (2.9%)	0.00852	0.0114	Reactome
Sarcoidosis exclusively					
VEGFA-VEGFR2 Signaling Pathway	236	12 (5.1%)	1.97E−08	2.76E−07	Wikipathways
MAPK signalling pathway	295	11 (3.7%)	1.79E−06	1.25E−05	KEGG
Diseases of signal transduction	248	10 (4.0%)	2.73E−06	1.27E−05	Reactome
Focal Adhesion-PI3K-Akt-mTOR-signaling	302	10 (3.3%)	1.55E−05	5.42E−05	Wikipathways
Nuclear Receptors Meta-Pathway	316	10 (3.2%)	2.29E−05	6.41E−05	Wikipathways
Signaling by Receptor Tyrosine Kinases	423	11 (2.6%)	5.30E−05	0.00012	Reactome
PI3K-Akt signalling pathway	354	10 (2.8%)	5.86E−05	0.00012	KEGG
Pathways in cancer	526	11 (2.1%)	0.00036	0.00063	KEGG
Signal Transduction	2647	29 (1.1%)	0.00065	0.00102	Reactome
Disease	510	10 (2.0%)	0.00108	0.00151	Reactome
JAK STAT pathway and regulation	310	9 (2.9%)	0.00011	0.00025	INOH
PI3K-Akt Signaling Pathway	340	9 (2.7%)	0.00023	0.00046	Wikipath
EGFR1	457	9(2.0%)	0.00187	0.00257	NEtPath

Input list were mapped in ConsensusPathDB. The total of genes identified in all granulomatous tissue in the three granulomatous diseases (250 DEG); then we mapped 87 DEG that were identified as exclusively is Sarcoidosis. We included pathways as defined by different pathway databases with a minimum overlap of 20 genes for the total number of dysregulated genes and a minimum overlap of 8 genes for the sarcoidosis gene set with a p value cut off 0.01

### Independent validation of disease-specific granuloma transcriptome results

We further validated our results using independent cohorts of microarray expression in lymph node and lung tissues from sarcoidosis and tuberculosis (Additional file 5: Fig. S1). Validation analysis in these microarrays data sets confirmed the dysregulation of 90 genes (FC > 2 FDR < 0.012) of the DEG identified in our panel. Among those dysregulated transcripts, we validated 46 in TB and 44 in sarcoidosis, 14 of them differentially expressed in lung, 30 in lymph nodes and 4 genes dysregulated in both tissues *ADAMST1, CXCL2, CXCL9, FABP4*. The direction of the expression defined by fold change was confirmed in all the dysregulated genes in the microarray data.

## Discussion

Sarcoidosis is a heterogeneous disease lacking validated clinical markers that allow differentiation from other granulomatous diseases or that predict the severity or level of organ involvement. In this study, we unveil the transcriptome profiles of cells comprising granulomas from sarcoidosis, CM, and TB. Using the HTG EdgeSeq oncology biomarker panel system to sequence FFPE biopsied tissues collected at the time of the diagnosis, we avoid limitations such as low RNA yields associated to FFPE extractions. We generated information to further define the molecular mechanisms that occur at the compartment level in sarcoidosis and compared to diseases that share a common histopathological hallmark.

Sarcoidosis granulomas showed differences in gene pathway expression between compartments potentially associated to disease progression. Lymph node granulomas exhibited clear immunological responses, involving leucocyte migration and neutrophil chemotaxis corresponding to early clinical stages observed in sarcoidosis. A structural regenerative response was observed in lung granulomas, notoriously marked by cell migration and angiogenesis, that appeared to correspond to lung remodeling stages observed in complicated and chronic sarcoidosis.

Only ten DEGs were common to both sarcoidosis lung granulomas and lymph nodes granulomas. *ADAMTS1, CXCL2, HSPB6, ITGA9, NPR1, NR4A1, CCL14, CXCL9, FABP4* and *NR1H3.* Interestingly *NR1H3*, a regulator of macrophage function and inflammation, and CXCL9 a chemokine ligand involved in T-cell trafficking, were upregulated in both granulomas independently of the tissue origin (FC > 2.6). However, *CXCL9* was also upregulated in the TB granulomas. Downregulated genes included FABP4, a fatty acid-binding protein associated with activation of proinflammatory macrophages in breast cancer, obesity and leukemia progression [39, 40]. FABP4, was previously proposed as sarcoidosis candidate gene [22, 41]. We identified that it was also significantly downregulated in TB. *CCL14*, a chemokine ligand associated with changes in intracellular calcium concentration in monocytes. Hypercalcemia associated to granulomatous diseases is commonly reported [42–44], the potential pathogenic role of *CXCL13* and *CXCL*14, both DEG in sarcoidosis and TB needs further investigation.

Despite similarities at the histological level, our analysis revealed sarcoidosis granulomas to be significantly divergent at the genomic level when compared to CM granulomas. TB and sarcoidosis granuloma profiles share stronger similarity at the transcriptional and pathway level. Interestingly, the number of DE genes in each comparison using the same thresholds varied significantly, indicating that the number of dysregulated genes does not correlate with the number of samples per comparison, or the size of the microdissection, suggesting that the granulomas are qualitatively distinct depending on the disease and tissue of origin. We identified only three shared dysregulated transcripts in CM, TB and sarcoidosis (lymph nodes); *CXCL13, CD27* and *SLAMF7*, a finding that points to diverse pathogenic mechanisms in granulomatous diseases that result in a similar histopathological conglomeration of CD4+ T cells surrounded by CD8+ T cells, fibroblasts and B cells. CXCL13 is a chemokine with key role in B cell migration by regulation of Ca++ influx [45], while SLAMF7 is a regulator of T lymphocyte development and function such as lytic activity and a modulator of B cell activation and memory [46]. Furthermore, CD27 regulates B-cell activation and immunoglobulin synthesis through the activation of NF-kappaB and MAPK8/JNK. Our microarray validation data corroborated *SLAMF7* DE but only in sarcoidosis lymph node granulomas.

The activation of Th1 cells capable of producing interferon (IFN)-γ is important in the immunological pathogenesis of the granuloma formation [47]. Interferon-inducible neutrophil-driven blood transcriptional signature was previously associated to TB and one of the two sarcoidosis profiles (weaker vs strong IFN-inducible profile), with a higher abundance and expression in tuberculosis [48]. Our panel included probes for IFN related genes such as IFNGR1, IRF1, IFI27, IFIT2, IFNA2, IFNAR1, IFNB1, IFNG and IFN regulatory related genes; IRF2, IRF3, IRF4, IRF5, IRF7 and IRF8. Only IFI27 was significantly upregulated in TB (p 0.0000678 FC > 6) but didn’t achieve significance in Sarcoidosis. IFI27 is involved in type-I interferon-induced apoptosis characterized by a rapid release of cytochrome C, related pathways include innate immune system and interferon gamma signaling. The IFN immune related pathways that contribute to sarcoidosis etiopathogenesis are reported to be exacerbated by immunotherapy with IFN alpha [49–51]. Further investigation is needed to explain the pathogenic effect of the observed differences at the gene expression level.

This study also served to demonstrate the lack of specificity of prior suggested potential sarcoidosis genomic markers due to common dysregulation in fungal or mycobacterium granulomas. For example, *CXCL9*, a chemoattractant for lymphocytes previously proposed as a marker of sarcoidosis severity [26], was also over-expressed in TB and is also dysregulated in Beryllium disease [27]. On the other hand, we confirmed downregulation of *NOTCH4* expression to be exclusively present in lung sarcoidosis. NOTCH signaling is initiated after activation of toll-like receptors in macrophages and it has been previously reported that Notch receptor ligand Dll4 is expressed in cutaneous sarcoidosis granulomas [52]. Supporting this association, SNPs in *NOTCH4* have been reported as a sarcoidosis-associated locus in genome-wide associated study in African Americans [1].

We have previously generated a 20-sarcoidosis gene signature by microarray in peripheral blood from PBMCs [33]. We now confirm three genes (*SERTAD1, HBEGF, KLRB1*) as DEGs by oncopanel profiling. *KLRB1* expression was also downregulated in TB and *SERTAD1* was significantly downregulated in CM. Therefore, these two genes appear to be part of common process in granulomatous diseases. In contrast, HBEGF, a heparin-binding epidermal growth factor, was down-regulated in sarcoidosis lung granulomas (FC − 2.36, p = 0.0016) and was not dysregulated in CM or TB. HBEGF is involved in epithelialization, wound contraction and angiogenesis with an active pro-inflammatory role in skin and lung alveolar regeneration [24], pneumonitis and early stages of systemic sclerosis [25]. Importantly, within the sarcoidosis PBMC molecular signature, *HBEGF* expression was up-regulated in contrast to the observed down-regulation in granuloma tissues. This variation in gene expression across tissues may be explained by either heritability at *cis* loci, by *trans* regulation across tissues [53, 54] or by epigenetic mechanisms. Our previously conducted epigenetic studies in sarcoidosis identified IL6ST and *STAB1* as part of a miRNA-derived gene signature for complicated sarcoidosis [5]. In the current study, *IL6ST* was significantly dysregulated only in TB granulomas (FC − 1.42 and p 0.0008), while *STAB1* gene was significantly downregulated in lymph nodes in sarcoidosis (FC − 1.64, p 0.0014). *STAB1* codes for a transmembrane receptor that is expressed in endothelial cells and lymph nodes, with functions in angiogenesis, lymphocyte homing and cell adhesion which are key aspects in the chronicity of the granuloma.

Leveraging the power derived from publicly available microarray data sets from independent cohorts, we successfully validated *ADAMST1, CXCL2 and FABP4*, that were identified DEGs by the oncopanel. These three genomic markers were present exclusively in sarcoidosis in both lung and lymph node. NPR1, Natriuretic Peptide Receptor 1, is associated with cardiac hypertrophy and fibrosis [55] and has not been previously associated to sarcoidosis, was present in the lung and lymph node transcriptomic profiles and was also validated in microarray datasets but only in sarcoidosis granulomas from lymph node. *ADAMTS1,* gene encodes a disintegrin and metalloproteinase with thrombospondin motif associated with various inflammatory processes and a potentially important regulator of pathogenic granuloma formation in sarcoidosis in both compartments.

There are some limitations of the current study. One is related to RNA from FFPE tissues, despite the use of quantitative nuclease protection chemistry that enabled extraction-free quantitation of mRNA, RNA quantity and quality are limited in FFPE compared to cryopreserved tissues. Other limitations include the use of a sequencing panel and not whole genome sequencing, that limited number of gene probes and the expression levels of some genes was undetected. Our comparisons were against tissues and not immune cells, that and the difference in platforms (RNAseq vs microarrays) can explain the limited overlap to already published data sets. Last, the only gene expression data available in CM was microarray data in mice strains limiting the validation of our results in CM. Despite the observed differences at the pathway level in sarcoidosis granulomas at lung and lymph node that indicate disease progression, further investigations are needed to corroborate the expression of these genes in sarcoidosis heterogeneous subphenotypes since limited clinical data was available to establish these correlations.

## Conclusions

Sarcoidosis is a complex systemic disease of unknown cause with clinical care hampered by the paucity of available tools for diagnosis and prognosis. Our study is the first to identify RNAseq-derived transcriptome differences in granulomas from lung and lymph nodes of sarcoidosis subjects compared to CM and TB. Our data suggest that immunological signaling related-pathways distinguishes sarcoidosis from other granulomatous diseases and fills a crucial gap in knowledge of gene expression within granulomas. This study corroborates previous genomic markers suggested for sarcoidosis such as STAB1, HBEGF, FABP4 and NOTCH4, with HBEGF and NOTCH4 only expressed in lung granulomas. We have identified new genomic markers for sarcoidosis, ADAMTS1, CXCL2, and NPR1. This relevant translational information in sarcoidosis and other two histopathological similar infections provides important information for the future development of meaningful specific genomic-derived biomarkers for sarcoidosis diagnosis and prognosis.

## Supplementary information

**Additional file 1: Table S1.** Oncopanel gene list. List of the complete biomarker panel genes and the biological pathways assayed.

**Additional file 2: Table S2.** Sarcoidosis clinical phenotype. Scadding stage based on radiographic imaging which consist of Stage I: hilar enlargement only; Stage II: hilar enlargement plus interstitial lung disease; Stage III: interstitial lung disease and Stage IV: lung fibrosis. Lung involvement and other organs affected based on clinical data. All subjects have at least one reported clinical symptom related to lung involvement (shortness of breath, cough). NA. Information not available.

**Additional file 3: Table S3.** Microarrays data sets used for validation of the DEG identified by the oncopanel.

**Additional file 4: Table S4.** Sarcoidosis, TB and CM top common dysregulated genes. Common DEGs in granulomas derived this oncopanel in lung sarcoidosis and CM and in granulomas derived from lymph nodes from TB and Sarcoidosis.

**Additional file 5: Fig. S1.** Microarray validation. Comparison of granuloma DEGs identified by the oncopanel to microarray datasets. Heat maps representing expression profiles of validated gene sets in A) Sarcoidosis Lung 14 transcripts validated, B) Sarcoidosis Lymph nodes, 30 transcripts validated and C). TB lymph nodes, 46 transcripts validated. Transcripts differentially regulated showed a fold change ±2 & FDR<0.01.

## Data Availability

Databases generated from this study are available at GEO: https://www.ncbi.nlm.nih.gov/geo/query/acc.cgi?acc=GSE157671

## Abbreviations

DEGs: Differentially Expressed Genes
DE: Differential Expression
TB: Tuberculosis
CM: Coccidioidomycosis
GEO: Gene Expression Omnibus
FC: Fold Change
FFPE: Formalin-fixed, Paraffin-embedded
FDR: False Discovery Rate
