# Supplementary material for: Differential transcriptomics in sarcoidosis lung and lymph node granulomas with comparisons to pathogen-specific granulomas
Source: Respir Res. 2020 Dec 4;21:321. doi: 10.1186/s12931-020-01537-3 (PMC7716494; doi:10.1186/s12931-020-01537-3)
Supplement: Supplementary file 2 — Additional file 2: Table S2. Sarcoidosis clinical phenotype. Scadding stage based on radiographic imaging which consist of Stage I: hilar enlargement only; Stage II: hilar enlargement plus interstitial lung disease; Stage III: interstitial lung disease and Stage IV: lung fibrosis. Lung involvement and other organs affected based on clinical data. All subjects have at least one reported clinical symptom related to lung involvement (shortness of breath, cough). NA. Information not available. [file 12931_2020_1537_MOESM2_ESM.pdf]

Table S2. Sarcoidosis clinical phenotype

Record ID	Granuloma origin	Scadding Stage	Lung involment	Other organ affected
lungSarc01	Lung	IV	Yes	No
lungSarc02	Lung	II	Yes	No
lungSarc03	Lung	NA	Yes	Heart
lungSarc07	Lung	IV	Yes	No
lungSarc08	Lung	III	Yes	No
lungSarc04	Lung	III	Yes	Eye, skin
lymphSarc03	Lymph node	IV	Yes	No
lymphSarc02	Lymph node	II	Yes	No
lymphSarc13	Lymph node	III	Yes	Bone
lymphSarc05	Lymph node	II	Yes	Peripheral nerve
lymphSarc01	Lymph node	III	Yes	Heart
lymphSarc08	Lymph node	NA	Yes	NA
lymphSarc06	Lymph node	II	Yes	No
lymphSarc10	Lymph node	NA	Yes	No
lymphSarc09	Lymph node	NA	Yes	NA
lymphSarc07	Lymph node	II	Yes	No
lymphSarc12	Lymph node	NA	Yes	No
lymphSarc14	Lymph node	II	Yes	Bone
