# Supplementary material for: Differential transcriptomics in sarcoidosis lung and lymph node granulomas with comparisons to pathogen-specific granulomas
Source: Respir Res. 2020 Dec 4;21:321. doi: 10.1186/s12931-020-01537-3 (PMC7716494; doi:10.1186/s12931-020-01537-3)
Supplement: Supplementary file 3 — Additional file 3: Table S3. Microarrays data sets used for validation of the DEG identified by the oncopanel. [file 12931_2020_1537_MOESM3_ESM.pdf]

Table S3. Microarrays data sets

Dataset	Diagnosis	Species	Tissue	Array
**GSE16538**	Sarcoidosis	H.sapiens	Lung	Affymetrix array HU-U133A_2
**Unpublished**	Sarcoidosis	H.sapiens	Lymph node	Affymetrix array HU-U133A_2
**GSE63548**	Tuberculosis	H.sapiens	Lymph node	Illumina HumanHT-12 V4.0
