# Supplementary material for: Differential transcriptomics in sarcoidosis lung and lymph node granulomas with comparisons to pathogen-specific granulomas
Source: Respir Res. 2020 Dec 4;21:321. doi: 10.1186/s12931-020-01537-3 (PMC7716494; doi:10.1186/s12931-020-01537-3)
Supplement: Supplementary file 4 — Additional file 4: Table S4. Sarcoidosis, TB and CM top common dysregulated genes. Common DEGs in granulomas derived this oncopanel in lung sarcoidosis and CM and in granulomas derived from lymph nodes from TB and Sarcoidosis. [file 12931_2020_1537_MOESM4_ESM.pdf]

Genes	Tuberculosis				Sarcoidosis (lymph node)			
	Log2 FC	FC	p value	p Adj. value	Log2 FC	FC	p value	p Adj. value
GPNMB	3.9	14.93	3.24E-05	0.00359	3.51	11.39	4.52E-07	0.001
CHI3L1	4.58	23.92	7.45E-08	0.000182	5.54	46.53	8.23E-07	0.001
APBB1	-1.98	-3.94	0.000166	0.00864	-1.72	-3.29	1.67E-05	0.00818
GM2A	2.33	5.03	0.000277	0.0125	2.48	5.58	1.99E-05	0.00818
LEF1	-3.12	-8.69	3.77E-05	0.00383	-1.9	-3.73	2.41E-05	0.00818
LGALS3	2.38	5.21	0.00016	0.00864	1.48	2.79	3.42E-05	0.00834
CEBPA	1.65	3.14	0.0014	0.0279	1.91	3.76	4.49E-05	0.00995
CCR7	-3.08	-8.46	4.79E-05	0.00427	-1.62	-3.07	0.000143	0.0183
CCL18	3.69	12.91	6.84E-07	0.000539	1.55	2.93	0.000204	0.0208
CTSD	3.38	10.41	5.01E-05	0.00427	2.25	4.76	0.00022	0.0208
KIT	-2.07	-4.2	0.00455	0.0499	-1.9	-3.73	0.000232	0.021
BCAT1	1.71	3.27	0.000159	0.00864	1.7	3.25	0.00026	0.0226
TNFRSF21	-1.62	-3.07	0.00343	0.0437	-2.09	-4.26	0.000282	0.0237
SLAMF7	2.02	4.06	0.002	0.0345	1.91	3.76	0.000307	0.0245
FOXP1	-1.98	-3.94	5.07E-05	0.00427	-1.46	-2.75	0.000311	0.0245
TCF7	-3.6	-13.93	4.45E-05	0.00418	-1.85	-3.61	0.000468	0.0292
MMP14	2.61	6.11	1.09E-05	0.00221	1.66	3.16	0.000625	0.0331
PLA1A	-1.91	-3.76	0.00208	0.0346	-1.43	-2.69	0.000845	0.0374
TNFAIP2	2.03	4.08	0.000164	0.00864	1.8	3.48	0.000869	0.0374
FBP1	3.77	13.64	4.04E-05	0.00394	4.67	25.46	0.000902	0.0374
CTSB	2.39	5.24	8.22E-05	0.0057	1.47	2.77	0.00125	0.0438
ETS1	-2.35	-5.1	2.03E-05	0.00268	-1.62	-3.07	0.00168	0.0475
CXCR4	-2.52	-5.74	0.00105	0.0244	-1.77	-3.41	0.00181	0.0484
LYVE1	-2.05	-4.14	0.00283	0.0399	-2.03	-4.08	0.00206	0.0517
IL7R	-2.8	-6.96	1.63E-05	0.00265	-1.63	-3.1	0.00219	0.0518
CSTB	2.33	5.03	0.000798	0.0221	1.86	3.63	0.00231	0.0519
CD209	-2.67	-6.36	1.76E-05	0.00268	-2.06	-4.17	0.00235	0.0519
PLAU	1.75	3.36	0.000384	0.0146	1.4	2.64	0.00235	0.0519
NUPR1	2.51	5.7	0.000142	0.00807	2.06	4.17	0.00274	0.0556
ADRA1B	-2.8	-6.96	0.00365	0.0438	-1.61	-3.05	0.00311	0.0598
SLC16A3	1.98	3.94	0.000307	0.0131	1.5	2.83	0.00339	0.0628
CD27	-3.36	-10.27	0.00451	0.0497	-1.49	-2.81	0.00403	0.0692
GADD45G	2.42	5.35	0.00356	0.0438	2.25	4.76	0.00506	0.0785
AQP9	1.96	3.89	0.000797	0.0221	1.04	2.06	0.00853	0.0961
CXCL13	-3.32	-9.99	9.85E-05	0.00616	-1.54	-2.91	0.00174	0.048
CCL14	-2.55	-5.86	0.000252	0.0116	-2.48	-5.58	0.00364	0.0657
NR1H3	1.81	3.51	0.000349	0.0139	2.06	4.17	0.000208	0.0208
CXCL9	3.3	9.85	7.04E-06	0.00172	2.69	6.45	3.21E-05	0.00834
FABP4	-4.29	-19.56	0.000239	0.0114	-3.8	-13.93	2.17E-05	0.00818

Genes	Sarcoidosis (lung)	Valley Fever

	Log2 FC	FC	p value	p Adj. value	Log2 FC	FC	p value	p Adj. value
CSF3	-4.23	-18.77	0.0003	0.0684	-2.8	-6.96	0.0018	0.519
SERTAD1	-3.19	-9.13	0.0009	0.0811	-1.47	-2.77	0.0048	0.519
MMP9	4.94	30.7	0.0011	0.0815	2.34	5.06	0.0006	0.476
CCL19	2.92	7.57	0.0018	0.0887	2.26	4.79	0.0008	0.476
BCL3	-1.74	-3.34	0.0025	0.0887	-1.62	-3.07	0.0016	0.519
AREG	-2.9463	-7.7079	0.0049	0.1077	-2.7564	-6.7572	0.0059	0.518
PTGS	-2.1153	-4.3327	0.0016	0.0854	-1.3984	-2.6362	0.0046	0.5189
F3	-1.4035	-2.6453	0.0023	0.0887	-1.3154	-2.4887	0.0089	0.5872
