# Supplementary material for: Differential transcriptomics in sarcoidosis lung and lymph node granulomas with comparisons to pathogen-specific granulomas
Source: Respir Res. 2020 Dec 4;21:321. doi: 10.1186/s12931-020-01537-3 (PMC7716494; doi:10.1186/s12931-020-01537-3)
Supplement: Supplementary file 5 — Additional file 5: Fig. S1. Microarray validation. Comparison of granuloma DEGs identified by the oncopanel to microarray datasets. Heat maps representing expression profiles of validated gene sets in A) Sarcoidosis Lung 14 transcripts validated, B) Sarcoidosis Lymph nodes, 30 transcripts validated and C). TB lymph nodes, 46 transcripts validated. Transcripts differentially regulated showed a fold change ±2 & FDR<0.01. [file 12931_2020_1537_MOESM5_ESM.pdf]

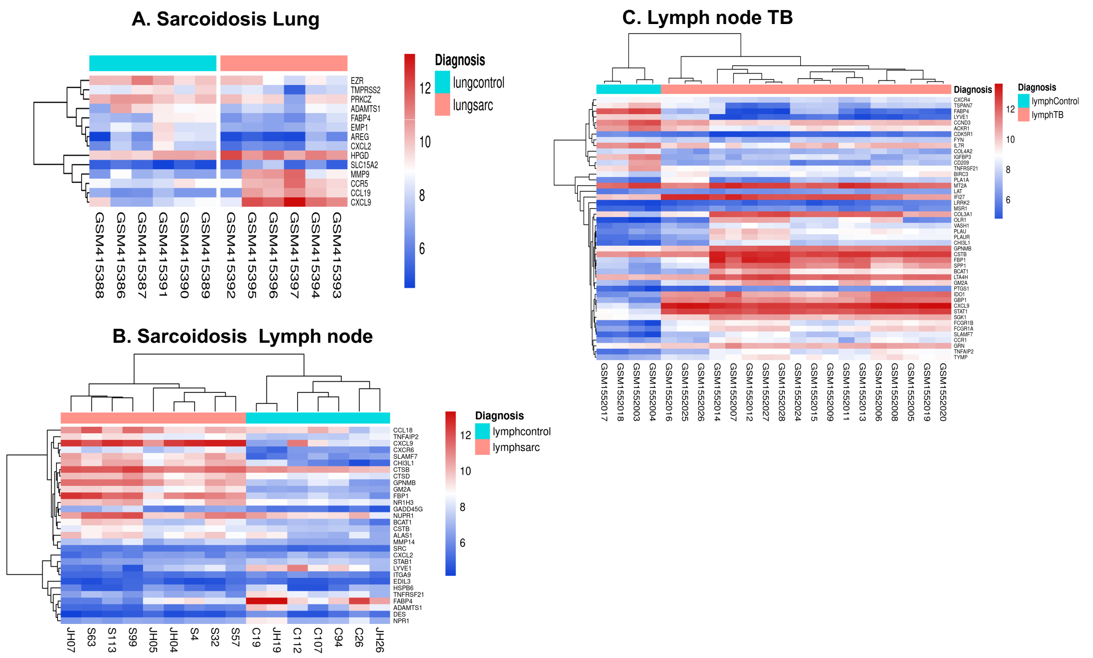
A. Sarcoidosis Lung
EZR
TMPRSS2
PRKCZ
ADAMTS1
FABP4
EMP1
AREG
CXCL2
HPGD
SLC15A2
MMP9
CCR5
CCL19
CXCL9
GSM415388
GSM415386
GSM415387
GSM415391
GSM415390
GSM415389
GSM415392
GSM415395
GSM415396
GSM415397
GSM415394
GSM415393
12
10
8
6
Diagnosis
lungcontrol
lungsarc
B. Sarcoidosis Lymph node
CCL18
TNFAIP2
CXCL9
CXCR6
SLAMF7
CHI3L1
CTSB
CTSD
GPNMB
GM2A
FBP1
NR1H3
GADD45G
NUPR1
BCAT1
CSTB
ALAS1
MMP14
SRC
CXCL2
STAB1
LYVE1
ITGA9
EDIL3
HSPB6
TNFRSF21
FABP4
ADAMTS1
DES
NPR1
JH07
S63
S113
S99
JH05
JH04
S4
S32
S57
C19
JH19
C112
C107
C94
C26
JH26
12
10
8
6
Diagnosis
lymphcontrol
lymphsarc
C. Lymph node TB
Diagnosis
CXCR4
TSPAN7
FABP4
LYVE1
CCND3
ACKR1
CDK5R1
FYN
IL7R
COL4A2
IGFBP3
CD209
TNFRSF21
BIRC3
PLA1A
MT2A
LAT
IFI27
LRRK2
MSR1
COL3A1
OLR1
VASH1
PLAU
PLAUR
CHI3L1
GPNMB
CSTB
FBP1
SPP1
BCAT1
LTA4H
GM2A
PTGS1
IDO1
GBP1
CXCL9
STAT1
SGK1
FCGR1B
FCGR1A
SLAMF7
CCR1
GRN
TNFAIP2
TYMP
GSM1552017
GSM1552018
GSM1552003
GSM1552004
GSM1552016
GSM1552025
GSM1552026
GSM1552014
GSM1552007
GSM1552012
GSM1552027
GSM1552028
GSM1552024
GSM1552015
GSM1552009
GSM1552011
GSM1552013
GSM1552006
GSM1552008
GSM1552005
GSM1552019
GSM1552020
12
10
8
6
Diagnosis
lymphControl
lymphTB
