# Supplementary material for: Differential transcriptomics in sarcoidosis lung and lymph node granulomas with comparisons to pathogen-specific granulomas
Source: Respir Res. 2020 Dec 4;21:321. doi: 10.1186/s12931-020-01537-3 (PMC7716494; doi:10.1186/s12931-020-01537-3)
Supplement: Supplementary file 1 — Additional file 1: Table S1. Oncopanel gene list. List of the complete biomarker panel genes and the biological pathways assayed. [file 12931_2020_1537_MOESM1_ESM.pdf]

Adhesion										
ADGRE5	ALCAM	CDH1	CDH5	CLEC4A						
CLEC4C	CLEC5A	CLEC6A	CLEC7A	DST						
EPCAM	FN1	IBSP	ICAM1	ICAM2						
ICAM3	ICAM4	ITGA1	ITGA2	ITGA4						
ITGA5	ITGA6	ITGAL	ITGAM	ITGB1						
ITGB2	ITGB3	ITGB4	L1CAM	LGALS1						
LGALS3	LGALS9	MBL2	MUC1	NCAM1						
PVR	SELE	SVIL	THBS1	THY1						
Apoptosis										
APAF1	BCL10	BCL2								
BCL2L1	BID	BIRC5								
CARD11	CASP1	CASP10								
CASP3	CASP8	DDX58								
FADD	NLRP3	NOD1								
NOD2	SSX1	TIMP1								
TP53	TRAF2	TRAF3								
TRAF6										
B-Cell	Function									
BLNK	BTK	BTLA	CD19							
CD79A	EBF4	FAS	CD22							
FASLG	FCER2	IKBKB	CD24							
IKBKG	MS4A1	PAX5								
RAG1	SLAMF1	SLAMF6								
SLAMF7	SPI1	SPN								
Cell	Cycle									
ABL1	ABL2	ATF1								
ATF2	ATM	AXL								
BATF	BAX	CCND1								
CCND3	CDK1	CDKN1A								
CDKN1B	EGR1	NCL								
REL	RELA	RELB								
SSX1	TBK1	TBX21								
TIMP1	TP53	TTK								
Cell	Signaling									
ADORA2A	AKT3	BCL6	CEBPB	CIITA	CREB1	CREB5	CREBBP	DUSP4	DUSP6	ELK1
EP300	ETS1	FLT3	FOS	GATA3	HES1	HES5	HEY1	HEY2	HEYL	HMGB1
MAF	MAP2K1	MAP2K2	MAP2K4	MAP2K7	MAP3K1	MAP3K5	MAPK1	MAPK11	MAPK14	MAPK3
MAPK8	MAPKAPK2	MEF2C	MTDH	MUC1	MYC	NFATC1	NFATC3	NFATC4	NFKB1	NFKB2
NFKBIA	NOTCH1	PIK3CD	PIK3CG	POU2F2	PRKCD	PRKCE	PTEN	PYCARD	RIPK2	RNF4
RORC	RUNX1	RUNX3	S100B	SEMA4D	SMAD2	SMAD3	SOCS1	SOCS3	SPINK5	STAT1
STAT2	STAT3	STAT4	STAT5B	STAT6	SYK	TAL1	TCF7	TYK2		
Chemokine										
CCL1	CCL11	CCL13	CCL14	CCL15	CCL16	CCL17	CCL18			
CCL19	CCL2	CCL20	CCL21	CCL22	CCL23	CCL24	CCL25			
CCL26	CCL27	CCL28	CCL3	CCL4	CCL5	CCL7	CCL8			
CCR1	CCR2	CCR3	CCR4	CCR5	CCR6	CCR7	CCR9			
CCRL2	CKLF	CMKLR1	CX3CL1	CX3CR1	CXCL1	CXCL10	CXCL11			
CXCL12	CXCL13	CXCL14	CXCL16	CXCL2	CXCL3	CXCL5	CXCL6			
CXCL8	CXCL9	CXCR1	CXCR2	CXCR3	CXCR4	CXCR5	CXCR6			
MST1R	PPBP	XCL2	XCR1							
Complement										
C1QA	C3	C3AR1	C5	CD55	CD59	NRP1				
Cytokine										
ADAM17	CSF1	CSF1R	CSF2	CSF3	CSF3R	IFNGR1	IRF1	LTB	LTBP1	
IFI27	IFIT2	IFNAFamily	IFNA2	IFNAR1	IFNB1	IFNG	LTA	SOCS1	SOCS3	
IRF2	IRF3	IRF4	IRF5	IRF7	IRF8	LIF	PTGS2	TNFRSF11B	TNFRSF12A	
LTBR	MIF	MNDA	MX1	OSM	PDGFC	PDGFRB	TNFRSF11A	TNFRSF8	TNFRSF9	
SPP1	TGFB1	TGFB2	TNF	TNFAIP3	TNFRSF10B	TNFRSF10C	TNFRSF4	TNFSF4	TNFSF8	
TNFRSF13B	TNFRSF13C	TNFRSF14	TNFRSF17	TNFRSF18	TNFRSF1A	TNFRSF1B	TNFSF18			
TNFSF10	TNFSF11	TNFSF12	TNFSF13	TNFSF13B	TNFSF14	TNFSF15				
TNFSF9	VEGFA	VEGFC	ENG	FLT3LG	GPI	IFI16				
Housekeeper										
ACTB	ATP5F1	DDX5	EEF1G							
EIF2A	GAPDH									
HMOX1	HSPA1A									
OAZ1	PPIA									
RPL38	RPL6									
RPS7	SOD1									
TBP	YWHAZ									
Immune	Function									
ADA	AICDA	ALOX5	APP	CAMP	CD14	CYBB	DEFB1			
FCMR	GNLY	GZMA	GZMB							
GZMH	GZMK	GZMM	HLA-A							
HLA-B	HLA-DRA	HLA-E	IDO1							
IDO2	PLA2G7	PRF1	TAP1							
TAP2	TREM1	TREM2	TXNIP							
Interleukin	Signaling									
IL10	IL10RA	IL10RB	IL11	IL11RA	IL12A	IL12B	IL12RB2			
IL18	IL18BP	IL19	IL1A	IL1B	IL1R1	IL1R2	IL13	IL13RA2		
IL1RAP	IL1RN	IL2	IL20	IL20RA	IL20RB	IL21	IL16	IL17A	IL17F	
IL22	IL22RA1	IL22RA2	IL23A	IL23R	IL24	IL27	IL15			
IL2RA	IL2RB	IL2RG	IL3	IL32	IL33	IL3RA				
IL4	IL4R	IL5	IL5RA	IL6	IL6R	IL6ST				
IL7	IL7R	IL9	IRAK1	IRAK2						
Lineage	Marker									
CD14	CD160	CD19	CD1A	CD2	CD200	CD209	CD22			
CD33	CD34	CD38	CD3D	CD4	CD40	CD40LG	CD24			
CD44	CD47	CD5	CD52	CD55	CD59	CD63	CD27			
CD68	CD69	CD70	CD74	CD79A	CD80	CD83	CD244			
CD86	CD8A	CD99	DPP4	ENTPD1	FCER2	FCGR1A	CD276			
FCGR1B	FCGR3A	FOXP3	HAVCR2	LYN	MME	POU2AF1				
TBX21	TFRC						CD28			
NK	Function									
CD244	FCGR3A	KIR2DS1	KIR3DL3	KLRB1	KLRD1	KLRG1				
Other										
BMI1	CD200	CES1	COL3A1							
CTRC	IRGM	MAGEA1	MAGEA3							
MAGEA4	MAGEB2	MAGEC1	MAGEC2							
MMP12	MMP9	MSH2	MSH3							
MSH4	MSH5	MSH6	MSR1							
PLAU	PLAUR	PPARG	PRAME							
SERPINA1	SERPINB2	SERPINE1	TFRC							
Proteosome										
CHUK	CTSH									
CTSL	CTSS									
CYLD	ISG15									
PSMD7	RBX1									
RNF149	UBE2T									
UBE3A										
T-Cell	Function									
BTLA	CD1A	CD3D	CD4	CD274	CD276					
CD70	CD80	CD2	CD27	CD99	CTLA4					
DPP4	EOMES	CD86	CD8A	GNLY	ICOS					
ICOSLG	LAG3	FOXP3	GATA3	NFATC3	NFATC4					
PDCD1	PDCD1LG2	LAT	NFATC1	SLAMF1	SLAMF6					
SLAMF7	SPN	PTPRC	RAG1	TCL1B	CD69					
	CD28	SUSD3	TCF7	CD40LG	CD40					
Toll-Like	Receptors									
LY96	MYD88									
TLR1	TLR2	TICAM1	TIRAP							
TLR3	TLR4									
TLR5	TLR6									
TLR7	TLR8									
TLR9	TOLLIP									
Transporter										
A2M	ABCB1									
ABCC2	ABCG2	ABCB11								
ATG12	ATG16L1	ANXA1								
ATG7	CRP	ATG5								
MERTK	NT5E	FYN								
SLC11A1	SLC25A3	PECAM1								
TAP2		TAP1								
Tumor	Necrosis	Factor								
TNFRSF10B	TNFRSF10C	TNFRSF11A	TNFRSF12A							
FADD	TNF	TNFAIP3	TNFRSF11B							
TNFRSF18	TNFRSF1A	TNFRSF1B	TNFRSF4							
TNFRSF8	TNFRSF9	TNFSF10	TNFSF11							
TNFSF12	TNFSF13	TNFSF13B	TNFSF14							
TNFSF15	TNFSF18	TNFSF4	TNFSF8							
TNFSF9	TRAF2	TRAF3	TRAF6							
